# Genetic Factors Associated with Response to Vitamin E Treatment in NAFLD

**DOI:** 10.3390/antiox11071284

**Published:** 2022-06-28

**Authors:** Mehtap Civelek, Maren C. Podszun

**Affiliations:** Department of Nutrition and Dietetics, Institute of Nutritional Medicine, University of Hohenheim, 70599 Stuttgart, Germany; mehtap.civelek@uni-hohenheim.de

**Keywords:** vitamin E, NAFLD, response to treatment, genetic factors

## Abstract

Non-alcoholic fatty liver disease (NAFLD) is becoming the predominant liver disease worldwide, and vitamin E has been clinically shown to improve histological parameters in a subset of patients. In this narrative review, we investigate whether genetic factors may help to explain why some patients show histological improvements upon high-dose alpha-tocopherol (αT) treatment while others do not. In summary, we identified two factors that are associated with treatment response, including genetic variations in haptoglobin as well as fatty acid desaturase 1/2 (FADS1/FADS2). Other genetic variants such as in alpha-tocopherol transfer protein (αTTP), tocopherol associated protein (TAP), transmembrane 6 superfamily 2 (TM6SF2), cluster of differentiation 36 (CD36), and proteins involved in lipoprotein metabolism may also play a role, but have not yet been investigated in a clinical context. We propose to further validate these associations in larger populations, to then use them as a clinical tool to identify the subset of patients that will benefit the most from vitamin E supplementation.

## 1. Introduction

The excessive accumulation of hepatic lipids is the hallmark feature of non-alcoholic fatty liver disease (NAFLD), which is becoming the most common liver disorder in Western nations. Estimates for the global prevalence of the disease are as high as 24%, with some countries—including Germany, Finland, and the USA—reaching 30% and more [1,2]. As NAFLD is most often only diagnosed in the late stages, the actual prevalence may be even higher. NAFLD predominantly manifests in patients with obesity, insulin resistance, and dyslipidemia [3], although genetic factors, such as Patatin-like phospholipase domain-containing protein 3 (PNPLA3), play a role [2].

The diagnostic criteria for NAFLD are as follows: there must be evidence of steatosis either by imaging or histology as well as the absence of secondary causes of hepatic fat accumulation, such as alcohol consumption, steatogenic medication, genetic disorders, viral infections, autoimmune hepatitis, or hemochromatosis [4]. Histological evaluation of liver biopsies allows for the categorization of the disease as either non-alcoholic fatty liver (NAFL) or non-alcoholic steatohepatitis (NASH). NAFL is defined as the presence of hepatic steatosis in equal to or more than 5% of hepatocytes without injury, in the form of hepatocyte ballooning [4]. In NASH, on the other hand, in addition to steatosis (≥5%), there is ballooning and inflammation, and there may or may not be fibrosis visible [4]. To evaluate treatment efficacy in clinical trials, the NAFLD activity score (NAS) was developed [1]. NAS is the unweighted sum of the histopathological scores for steatosis (0–3), lobular inflammation (0–3), and ballooning (0–2), yielding a total that ranges from 0 to 8 [1]. Clinical trials also often specify NASH resolution as an outcome. This is defined as the absence of ballooning and minimal lobular inflammation (grade 1) without worsening of the stage of fibrosis, while steatosis can be present at any grade [5].

The underlying mechanism for the progression from NAFL to NASH is still unclear [6], and about 40% of all NAFLD patients [7,8] progress to NASH. The resulting liver injury in NASH can be severe enough to require a liver transplantation, and it is predicted that NASH is becoming the leading cause for liver transplantations [9]. Currently there are no drugs approved for the treatment of NAFLD, and the best treatment option is a lifestyle intervention to improve nutrition and increase physical activity to reduce bodyweight [10].

Oxidative stress plays an important role in the pathogenesis of NAFLD. Patients with NAFLD and especially NASH show higher levels of oxidized linoleic metabolites in the blood [11] as well as higher hepatic lipid peroxidation products compared with healthy controls [12,13,14]. Treatment with the antioxidative vitamin E—or more precisely, its major congener RRR-alpha-tocopherol (αT)—has improved liver enzymes, lobular inflammation, hepatocellular ballooning, and steatosis in clinical trials [15,16], as well as hepatic oxidative stress [14]. Furthermore, αT treatment is also beneficial in NASH and is currently the only known intervention for improving transplant-free survival [17].

Vitamin E was discovered 100 years ago as a dietary factor necessary for sustaining rat pregnancy by Katherine J. Bishop and Herbert M. Evans [18]. Vitamin E is a family of eight congeners, with either a saturated alkyl side chain (for the tocopherols) or an unsaturated isoprenoid side chain (tocotrienols) as well as a different methylation pattern at the chromanol ring (α,β,γ,δ). Although all eight congeners are absorbed, only αT is retained and incorporated into VLDL [19]. Patients with NAFLD have significantly lower αT plasma concentrations [20] than controls, likely due to the accumulation within hepatic lipid droplets [21].

Although vitamin E has been shown to be clinically effective, it is not effective in everyone. For example, in the Pioglitazone, vitamin E, or placebo for nonalcoholic steatohepatitis trial (PIVENS), only about 50% of the patients within the vitamin E group showed an improvement in histological parameters such as steatosis, lobular inflammation, and hepatocyte ballooning [22]. While the PIVENS trial only included patients without diabetes, a study by Bril et al. later confirmed the clinical efficacy of vitamin E in patients with diabetes [16]. The percentage of patients achieving NASH resolution was similar to the ones observed in PIVENS (Table A1), although the effect for the individual histological scores was less pronounced, possibly due to the smaller numbers enrolled in the study. In the TONIC trial, the effect of vitamin E on NASH was investigated in children and adolescents [23]. Similarly to the above mentioned trials, TONIC also showed a significant effect of vitamin E on NASH resolution.

Upon closer inspection of Table 1, one can appreciate the high rate of improvement for the histological parameters in the placebo group. This is a tremendous problem for the design of clinical trials investigating NAFLD and NASH. Factors associated with the placebo response are thought to include lifestyle changes of patients upon enrollment in a clinical trial, an uneven distribution of genetic polymorphisms that influence disease severity, and sampling variability due to the histological endpoints [24]. However, even when taking into account all these variabilities, which will occur in the treatment group as well, the question still remains of what determines whether a patient will show histological improvement upon vitamin E treatment or not. This is especially important as there is still debate on whether or not high doses of αT are considered to be safe [25,26,27], and potential side effects include a higher tendency for bleeding [28,29] as well as an increased risk for the development of prostate cancer in men [30]. From a risk perspective, it would thus be ideal to only supplement those who will likely benefit from the treatment. Therefore, the scope of this narrative review is to discuss and summarize genetic factors that may influence the response to vitamin E treatment in NAFLD patients. We hope our review will provide the groundwork to further investigate who might benefit the most from vitamin E supplementation in NAFLD.

## 2. Genetic Factors Associated with Treatment Response

Plasma levels during vitamin E supplementation have been associated with NASH resolution and general histological improvement in both the PIVENS as well as the TONIC cohort [31]. Counterintuitively, lower plasma αT levels were associated with better histological improvements. Lower plasma levels might reflect lower absorption, an increase in vitamin E metabolism, higher intrahepatic levels, or increased usage of αT as an antioxidant. The relationship between hepatic and circulating vitamin E is likely dependent on multiple factors and does not follow a linear correlation [32]. Differences in plasma, hepatic, or even subcellular vitamin E could all play an important role in explaining the observed variation in response to αT treatment in NAFLD. In the following subsection, we will briefly highlight major genetic factors associated with disease development and then discuss genetic factors associated with vitamin E plasma and serum levels as well as factors that influence levels upon supplementation. We will further discuss in detail the few genetic associations with treatment response to vitamin E in NAFLD. Unfortunately, association of genetic factors with treatment response are severely limited by the sample size. The best data comes from PIVENS and TONIC, with a maximum of 368 patients [31], and even those numbers are relatively small for genetic association studies. The data, summarized in Table A2, should thus be treated as exploratory. Ideally, promising candidates should be evaluated in sufficiently powered studies to then allow identification of patients who will benefit the most from vitamin E supplementation prior to initiation of treatment.

### 2.1. Genetic Factors Influencing Vitamin E Levels

#### 2.1.1. Genes Involved in Vitamin E Absorption and Hepatic Uptake

The intestinal uptake of vitamin E is mediated by scavenger receptor class B member 1 (SR-BI), cluster of differentiation 36 (CD36), and NPC1-like intracellular cholesterol transporter 1 (NPC1L1) [33,34,35]. Not surprisingly, SNPs in both SR-BI (rs11057830, “Exon-8”) as well as CD36 (rs1527479) have been associated with vitamin E levels. For SR-B1, as well as CD36, the minor alleles are associated with a decrease in vitamin E circulatory levels [36,37,38]. After enteral uptake, vitamin E is transported to the liver in chylomicrons, which are then taken up into the liver by LDL receptor-related proteins (LRP), which is a step that also involves hepatic lipase (LIPC) [39]. A SNP in LIPC (rs1800588) is associated with higher circulating vitamin E levels [40], which may be caused by a less efficient hepatic uptake of vitamin E from chylomicrons and a transfer of vitamin E from chylomicrons to other lipoproteins without hepatic involvement.

#### 2.1.2. Genes Involved in Hepatic Transport

The alpha-tocopherol transfer protein (αTTP) is predominantly expressed in the liver [41] and is crucial for the distribution of αT to extrahepatic tissues. Mutations in this gene cause the inability to transfer αT from the liver to VLDL, resulting in the disease Ataxia with Vitamin E Deficiency (AVED) [42]. There is a common SNP (rs6994076) in the promoter region of TTPA which significantly reduces promoter activity in vitro [43]. The effect of this SNP on plasma levels has presented conflicting findings. The T/T genotype of rs6994076 has been associated with lower αT tocopherol levels in the ATBC (α-tocopherol and ß-carotene cancer prevention) trial at baseline (*n* = 847, adjusted for serum cholesterol) as well as after supplementation (*n* = 381) [44]. Conversely, another study in patients with or without glaucoma showed the opposite association, namely higher αT levels with the T/T genotype (*n* = 500, unadjusted) [45]. Whether differences may be explained by the different populations or the use of either adjusted or unadjusted αT values remains to be elucidated. Furthermore, it is unknown whether the reduced promoter activity would actually lead to clinically significant changes in the levels of hepatic αTTP expression and whether this would be either associated with changes in disease severity or treatment response. In general, little is known about the role of αTTP in NAFLD.

Besides αTTP, intracellular transport of αT is also facilitated by the tocopherol-associated protein (TAP) [46,47]. The distribution of αT within the cell might be a critical factor for the response to vitamin E. In vitro, de novo lipogenesis is inhibited by αT through a non-transcriptional mechanism involving SREBP-1 cleavage within the endoplasmic reticulum [48]. It might thus be feasible that response to αT in NAFLD is dependent on the correct subcellular distribution of αT within hepatocytes. rs2299826 is a SNP within the TAP intronic region, and having two copies of the minor allele is associated with an increase in circulating αT [44]. Whether this is associated with a decrease in hepatic αT, NAFLD severity, or response to treatment is currently unknown.

#### 2.1.3. Genes Involved in Extrahepatic Transport

Hepatic export and systemic distribution of αT is mediated by VLDL. As previously mentioned, αT is intra-hepatically incorporated into nascent VLDL, likely through α-TTP. Factors influencing VLDL assembly or secretion can thus significantly alter plasma levels of αT. One such factor is APOA5, which is involved in VLDL secretion [49]. The minor allele of SNP rs662799 within ApoA5 is associated with significantly higher circulating triglycerides as well as vitamin E [50,51]. Not surprisingly, the association with vitamin E vanishes once αT is corrected for cholesterol or triglyceride levels. Similarly, an APOA4 SNP (rs675) was initially associated with αT, but the association did not remain statistically significant after adjusting for cholesterol [37]. Apo C-III S1/S2 genotype (SNP not referenced) is associated with increased cholesterol levels [52] as well as with circulating αT levels. In this case, the association remains significant even after adjustments for cholesterol, albeit only in women [53]. Alterations in lipoprotein metabolism and resulting changes in hepatic αT export may both alter local αT concentrations within the liver, although formal studies regarding response to αT treatment in NAFLD are lacking. 

#### 2.1.4. Genes Involved in vitamin E metabolism

Vitamin E metabolism is complex and involves multiple steps. The initial hydroxylation of the side chain is catabolized by the cytochrome P450 enzymes CYP3A4 and CYP4F2 [54]. The side chain is then successively shortened by omega and beta-oxidation within peroxisomes and mitochondria, resulting in the final degradation metabolites, carboxyethylhydroxychromanols (CEHC) [55]. While the human diet contains predominantly γT and less αT, αT is selectively retained in the body while other vitamin E forms are rapidly degraded to CEHC [56].

γ-CEHC was retrospectively determined in baseline plasma samples of the PIVENS trial, and lower baseline γ-CEHC levels—suggesting decreased CYP mediated metabolism—were associated with histological improvements in the liver upon treatment [57].

There is a common variant of CYP4F2 (rs2108622) that is associated with significantly higher αT plasma levels in patients with the T/T allele in clinical trials [31,36] and decreased metabolism of all tocopherols in vitro [58]. The association of rs2108622 with histological response to vitamin E treatment was retrospectively determined in the PIVENS and TONIC trial (*n* = 368) [31]. Neither trial showed an association of this SNP with histological improvement upon vitamin E treatment, although the effect on vitamin E metabolism was recapitulated. Patients with T/T genotype had significantly higher vitamin E levels during treatment in both the PIVENS and the TONIC trial at week 48 but not week 96 [31]. According to the authors, the declining effect with time may be an adaptive response of the body to prolonged high doses of αT. It is noteworthy that NASH resolution as well as general histological improvements in both PIVENS and TONIC were associated with lower αT levels. As mentioned above, CYP4F2 genotype was not associated with histological response. It cannot be ruled out that the association did not reach statistical significance due to the small sample size, and there is indeed an association of response with the investigated CYP4F2 SNP. Alternatively, patients with lower αT plasma levels upon treatment may have higher αT levels in the liver either due to their CYP4F2 genotype or other genetic factors involved in vitamin E metabolism, such as αTTP.

Overall, there is an association of CYP4F2 genotype (rs2108622) with vitamin E levels, but not with histological response to vitamin E treatment in NAFLD.

### 2.2. Genetic Factors Associated with NAFLD

Genetic factors play a prominent role in NAFLD pathogenesis and account at least partially for the variability of disease severity and progression [59]. Multiple Genome-wide association studies (GWASs) have identified genetic variants that are associated with hepatic lipid accumulation, liver enzymes, NAFLD development, and disease severity. The most prominent example, which has been confirmed in multiple cohorts and ethnicities, is a single nucleotide polymorphism (SNP) in the gene patatin-like phospholipase domain-containing 3 (PNPLA3, rs738409) [60,61,62,63,64,65]. This SNP is associated with increased hepatocyte fat content and a greater predisposition to progressive forms of NAFLD [60,66]. The frequency distribution of the minor allele (G) in different ethnicities is 49% in Hispanics, 23% in Europeans and 17% in African Americans [60]. PNPLA3 encodes a protein known as adiponutrin (ADPN), with lipolytic as well as lipogenic properties [67]. The lipase activity in vitro is predominantly against triglycerides and retinyl esters in hepatic stellate cells [68]. However, the minor allele of rs738409 is associated with the loss of hydrolyzing function, resulting in accumulation of lipid droplets in the hepatocytes [66]. The PNPLA3 SNP is associated not only with liver fat content, but also with hepatic inflammation, hepatic steatohepatitis, fibrosis, and cirrhosis, indicating that it plays a key role in the development of NAFLD [69].

Studies assessing the relationship between vitamin E response and PNPLA3 are limited, and there is no data available from the PIVENS/TONIC trial. In a small exploratory study, 38 NAFLD patients received either 150, 300, or 600 mg vitamin E three times a day for one year [70]. Vitamin E was effective in lowering liver enzymes, non-invasive fibrosis scores (FIB-4 index and aspartate aminotransferase-to-platelet index (APRI)), and liver stiffness. Although the numbers are very small, there was no difference in response by PNPLA3 genotype (CC/CG *n* = 19; GG = *n* = 19). Furthermore, a retrospective analysis of αT plasma levels in the ATBC study according to PNPLA3 genotype revealed no significant differences [71]. The current data suggest that PNPLA3 is unrelated to response to treatment or vitamin E levels in NAFLD, although this needs to be confirmed in a larger study.

Another important SNP associated with NAFLD is in transmembrane 6 superfamily member 2 (TM6SF2, rs58542926) [63,64,65,72]. The function of TM6SF2 is currently unknown. Silencing the gene in hepatocytes in vitro is associated with an increase in intracellular triglycerides and a decrease in triglyceride rich lipoprotein secretion [73]. Similarly, knockdown of TM6SF2 in mice decreases very low-density secretion (VLDL) from the liver by about 50% [72], indicating a pivotal role in lipoprotein metabolism.

In humans, variants of TM6SF2 are associated with circulating triglyceride levels as well as NAFLD disease risk [72,73]. The minor allele of the TM6SF2 SNP (rs58542926) is associated with higher liver fat, higher liver injury markers ALT, and lower circulating levels of triglycerides as well as well as low-density (LDL) cholesterol [72]. Since TM6SF2 seems to play an important role in VLDL secretion from the liver, it is not surprising that vitamin E levels are associated with the rs58542926 SNP [74]. Because αT is exported from the liver by VLDL, carriers of the minor allele have lower plasma αT levels. Whether this translates to higher hepatic levels and a possible benefit in terms of response to vitamin E treatment in NAFLD has not yet been investigated.

SNPs in TM6SF2 (rs58542926), PNPLA3 (rs738409), and MBOAT7 (rs641738) were investigated together in NAFLD patients receiving multiple supplements, including vitamin E (606 mg silybin-phospholipids complex, 20 µg vitamin D, and 30 mg of vitamin) [75]. Having either a mutation in one or multiple SNPs was associated with no response to treatment. It remains to be seen whether any of the individual SNPs are associated with the response to vitamin E treatment in NAFLD.

The fatty acid desaturase 1 (FADS1) is one of the rate-limiting enzymes in the polyunsaturated fatty acid (PUFA) desaturation pathway, and is specifically involved in catalyzing the conversion of dihomo-gamma-linolenic acid (DGLA) (20:3n-6) into arachidonic acid (AA) (20:4n-6) and eicosatetraenoic acid (20:4n-3) into eicosapentaenoic acid (EPA) (20:5n-3) and docosahexaenoic acid (DHA) (22:6n-3) [75]. FADS1 has been previously associated with steatosis as well as NAFLD disease severity [76,77]. In a small, pediatric pilot analysis of children with the FADS/FADS2 rs174576 minor allele (CA/AA, *n* = 10), the so-called low-function allele, there was a greater reduction in steatosis, fibrosis, and NAFLD activity score after the combination treatment with DHA, choline, and vitamin E compared to children with the major allele (CC, *n* = 9) [78]. Even after adjusting for the common SNPs in PNPLA3 (rs738409) and TM6SF2 (rs58542926), the association remained significant. Whether FADS1 genotype plays a role in the response to vitamin E alone or this result is mainly driven by DHA, and whether or not this effect can be recapitulated in larger studies and adults, remains to be elucidated.

Besides SNPs, other genetic variations, such as copy number variations, are associated with NAFLD. An example is Haptoglobin (Hp), an acute phase protein which is highly expressed in the liver, especially during tissue damage, inflammation, infection, trauma, or malignant proliferation [79]. Hp binds to free hemoglobin in the plasma with a high affinity [80]. Free hemoglobin is toxic due to the iron in heme, which can promote the generation of reactive hydroxyl radicals in the presence of H_2_O_2_, resulting in DNA damage [81]. After formation of a strong noncovalent complex, the α2-glycoprotein enables the clearance and degradation of hemoglobin via CD163 cell-surface receptors on hepatocytes, Kupffer cells, and tissue macrophages [82]. Aside from its antioxidant activity, Hp also has immunomodulatory effects, including the inhibition of prostaglandin synthesis or the promotion of a strong Th1 cell response [82]. These effects are dependent on the Hp genotype. There are three major genotypes of Hp, namely Hp 1-1, Hp 2-1, and Hp 2-2, with a known distribution in the Western population of 16%, 48%, and 36%, respectively [83]. Hp-1 has one copy and Hp-2 two copies of exon 3 and 4 [84] Hp 1-1 genotype confers better protection against oxidative stress as well as a greater Th1 response compared with the Hp 2-2 genotype [82]. In NAFLD, patients with Hp 2-2 genotype have higher steatosis, lobular inflammation, ballooning, and NAFLD activity (NAS) scores than other genotypes. Hp 2-2 genotype has further independently been associated with fibrosis [85]. 

Since vitamin E is an antioxidant and Hp has antioxidative properties, it is suggested that the histological response to vitamin E may be closely associated with Hp genotype [86]. In other words, patients with the Hp 2 allele, who tend to have more severe NAFLD and less antioxidative benefit from Hp, might benefit more from supplementation of vitamin E. Studies examining the relationship between vitamin E response and Hp phenotype are rare and are mostly limited to initial large-scale intervention studies (PIVENS, TONIC). However, the limited data seems to support the notion that Hp genotype may play a role in determining the response to vitamin E treatment in NAFLD. A post hoc analysis of data from the PIVENS and TONIC showed that patients with Hp 2-2 had a significant improvement in histological markers, NAFLD Activity Score (NAS), and liver enzymes after vitamin E treatment [83]. Patients with the Hp 1-1 genotype showed no improvement in any of the parameters, whereas patients with the Hp 2-1 or Hp 2-2 genotype showed improved liver enzymes and histology. This association needs to be confirmed in further studies. There is currently a clinical trial underway in China that will investigate response to vitamin E treatment by Hp genotype [86]. In summary, the data suggests that NAFLD patients with the Hp 2 allele, which have an increase in oxidative stress, might benefit the most from vitamin E supplementation. This would be in accordance with the assumption that the beneficial effects of vitamin E in NAFLD are mediated mainly by the antioxidative effect of vitamin E [14,48].

Association of other important genetic factors of NAFLD (such as hydroxysteroid 17-beta dehydrogenase 13 (HSD17B13, rs6834314, rs13118664) [62,63,64] and glucokinase regulator (GCKR, rs13118664, rs1260326) [61,62,63]) with vitamin E plasma, serum levels, or treatment response in NAFLD have not been determined yet.

## 3. Conclusions

**Figure 1:**
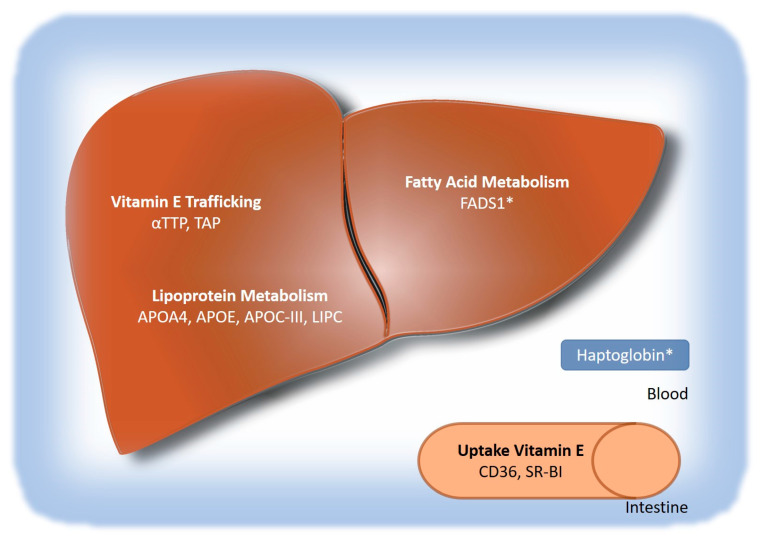
**Possible genetic factors associated with the response to vitamin E treatment in NAFLD.** Factors for which clinical evidence is available and an association has been reported are marked with an *. αTTP—α-tocopherol transfer protein; TAP—tocopherol-associated protein; APOA4—apolipoprotein A4; APOE—apolipoprotein E; APOC-III—apolipoprotein C-III; LIPC—hepatic lipase; FADS1—fatty acid desaturase 1; CD36—cluster of differentiation 36; SR-B1—scavenger receptor class B member 1.

There are multiple genetic factors that could explain the variability in response to vitamin E treatment in NAFLD, but conclusions are severely limited by the small sample sizes of the current trials (Figure 1). NASH resolution as well as histological improvement are associated with lower plasma αT levels, which indicates metabolism as a possible cause for the diverse treatment response. Interestingly, a variant in one of the major players in vitamin E metabolism, CYP4F2, is associated with plasma levels of αT but not with treatment response in NAFLD [31]. Besides CYP4F2, other genetic factors could be responsible for the differences in plasma levels, including proteins involved in absorption (CD36), intracellular trafficking (αTTP, TAP), and lipoprotein metabolism. They are all associated with plasma αT, but have not yet been investigated in terms of treatment response in NAFLD. Further studies should investigate whether or not genetic factors involved in the absorption, distribution, and metabolism of αT may offer an explanation for the differences in treatment response.

To the best of our knowledge, there are only two SNPs that have been associated with vitamin E treatment response in NAFLD. One of them is in FADS1/FADS2 (rs174576), which is associated with treatment response in pediatric NAFLD [78]. However, this association is only from a small pilot study wherein αT was administered as part of a multi- compound regime including choline and DHA. Therefore, further studies need to address whether this association remains when αT is given as a mono therapy and if it can also be recapitulated in adults. The most promising candidate so far is the haptoglobin genotype. Data from the PIVENS and TONIC trials point to a benefit of αT in patients that have two copies of exon 3 and 4 [83]. From a mechanistic point of view, this would be in line with the powerful hepatic antioxidative effect of αT [14], as this Hp genotype is associated with increased oxidative stress [82]. The results of the ongoing trial in China [86] will hopefully offer insight about whether or not the association can be replicated and whether haptoglobin genotype might be a valuable predictor for the clinical response to αT treatment in NAFLD.

Ideally, the response to vitamin E by genotype in NAFLD should be investigated in large-scale, multi-centered trials. By identifying those who benefit the most from vitamin E, unnecessary treatment delay for those who do not would be avoided. Furthermore, patients that will not benefit from the treatment will not be exposed to potential risks associated with high-dose αT treatment.

## Figures and Tables

**Table 1:** Percentage of patients (placebo vs. vitamin E) showing at least a one-point improvement in histological parameters. Statistical significance for the comparison vitamin E treatment to placebo: * *p* < 0.05.

	% Patients: Placebo vs. Vitamin E					
	**Improvement of Primary Outcome**	**Steatosis**	**Inflammation**	**Ballooning**	**NASH Resolution**	**Reference**
**PIVENS**	19 vs. 43 *	31 vs. 54 *	35 vs. 54 *	29 vs. 50 *	21 vs. 36	[22]
**TONIC**		27 vs. 31	20 vs. 22	10 vs. 22 *	28 vs. 58 *	[23]
**Bril et al.**		46 vs. 68	43 vs. 36	35 vs. 50	12 vs. 33 *	[16]

## Data Availability

All of the data is contained within the article.

## Appendix A

**Table A1:** Changes in mean score or ALT value from baseline in the vitamin E treatment group. Statistical significance for the comparison vitamin E treatment to placebo: * *p* < 0.05, ** *p* < 0.01, *** *p* < 0.001, + graphically estimated.

Numbers	Dose/Duration	Patient Characteristics	ALT Change or % Reduction from Baseline	Steatosis	Lobular Inflammation	Ballooning	NAS Score	Fibrosis	Ref
**80 placebo**, 80 vitamin E (PIVENS)	800 IU/d 96 weeks	Adults without diabetes, biopsy confirmed NASH (NAS score of at least 4, presence of ballooning)	−37 **	−0.7 ***	−0.6 **	−0.5 *	−1.9 ***	−0.3	[22]
58 placebo, 58 vitamin E (TONIC)	800 IU/d 96 weeks	Children (aged 8–17 years), biopsy confirmed NAFLD (>5% of hepatocytes showed macrovesicular fat)	Week 48: −44.5% *; Week 72: −44.2%; Week 96: −48.3%	−0.8	−0.4	−0.5 **	−1.8 *	−0.3	[23]
32 placebo/36 vitamin E	800 IU/d 72 weeks	Adults with diabetes, biopsy confirmed NASH (zone 3 macrovesicular steatosis with ballooning and lobular inflammation)	−20 *+	−1.0 *	−0.4	−0.5	not listed	−0.6	[16]
20 placebo/20 DHA-VE-CHO	250 mg of DHA, 39 UI of vitamin E, and 201 mg of choline; 48 weeks	Children (aged 4–16 years) with liver biopsy-proven NASH	−18.2 *	−0.8 *	−0.15	−0.75 **	−1.7 ***	0.17	[87]

**Table A2:** Genetic variations associated with α-tocopherol (αT) with and without supplementation as well as with treatment response in NAFLD. The minor allele frequency (MAF) given has been obtained from the ALFA project in the dbSNP database. Only variations with a MAF of at least 5% are included in this review. * indicates genes involved in splicing and not discussed in this review.

		MAF (Allele)	Effect of MA on αT Plasma/Serum Levels	References
**Polymorphisms associated with αT serum or plasma levels**				
APOA4	rs675	0.17 (A)	A lower	[37]
APOE	rs662799	0.09 (C)	T/T lower; T/C higher	[50,51]
Apo C-III	ApoC-III S1/S2	0.2 (C)	in women G/G lower	[53]
α-TTP	rs6994076	0.45 (A)	A/A higher; A/A lower	[44,45]
BUD13 *	rs28927680	0.06 (G)	not specified	[40]
	rs12292921	0.07 (A)	A higher	[36]
CD36	rs1527479	0.49 (T)	T/T lower	[38]
CYP4F2	rs2108622	0.29 (T)	T/T higher	[30,36]
LIPC	rs1800588	0.22 (T)	T higher	[40]
PNPLA3	rs738409	0.21 (G)	no association	[72]
SR-BI	rs11057830	0.15 (A)	A/A lower	[36]
SR-BI	exon 8	0.13 (C)	C/C higher	[37]
SLC30A8	rs13266634	0.29 (T)	not specified	[40]
SUGP1 *	rs10401969	0.08 (C)	not specified	[75]
TAP	rs2299826	0.11 (T)	T/T higher	[44]
TM6SF2	rs58542926	0.07 (T)	T lower	[75]
ZPR1 *	rs964184	0.15 (G)	G/G higher	[36,75]
**Polymorphisms associated with αT levels after supplementation**				
α-TTP	rs6994076	0.45 (A)	A/A higher on treatment	[44]
CYP4F2	rs2108622	0.29 (T)	T/T higher on treatment	[30,36]
**Polymorphisms and copy number variations associated with treatment response in NAFLD**				
CYP4F2	rs2108622	0.29 (T)	no association with treatment response	[30]
FADS1	rs174576	0.34 (A)	A associated with treatment response	[79]
Hp	CNV	0.16 (1/1)	Hp-2 allele associated with treatment response	[84]
